# Hall’s Journal of Health

**Published:** 1868-01

**Authors:** 


### HALL'S JOURNAL OF HEALTH.

THIS Periodical comes to us always freighted with things original, rare, spicy, sharp, and good. Every time we read a number of this excellent work, how much we wish it was in every family in the land, and by every member read, and its advice followed; what sickness, misery and woe would thus be averted. It aims to do what so many overlook, viz.: to stimulate people to live sharply up to their best intelligence, as well as to increase their stock of knowledge. Scarcely any make the best use of the knowledge they possess; their stock in trade is not used to the best advantage. We would say to all, if you wish an entertaining, interesting and instructive work upon health, the means of retaining, as well as of regaining it when lost; one that fearlessly strikes at error, popular or otherwise; take Hall's Journal of Health, not for a few numbers or a short time, but make a business of it, and when the habit is once formed, you will no more drop it than the regular meal. T.

